# Digital Anti-Aging Healthcare: An Overview of the Applications of Digital Technologies in Diet Management

**DOI:** 10.3390/jpm14030254

**Published:** 2024-02-27

**Authors:** Tagne Poupi Theodore Armand, Hee-Cheol Kim, Jung-In Kim

**Affiliations:** 1 Institute of Digital Anti-Aging Healthcare, Inje University, Gimhae 50834, Republic of Korea; poupiarmand2@gmail.com (T.P.T.A.); heeki@inje.ac.kr (H.-C.K.); 2 College of AI Convergence, u-AHRC, Inje University, Gimhae 50834, Republic of Korea

**Keywords:** anti-aging healthcare, diet management, digital technologies, healthy aging, personalized recommendations

## Abstract

Diet management has long been an important practice in healthcare, enabling individuals to get an insight into their nutrient intake, prevent diseases, and stay healthy. Traditional methods based on self-reporting, food diaries, and periodic assessments have been used for a long time to control dietary habits. These methods have shown limitations in accuracy, compliance, and real-time analysis. The rapid advancement of digital technologies has revolutionized healthcare, including the diet control landscape, allowing for innovative solutions to control dietary patterns and generate accurate and personalized recommendations. This study examines the potential of digital technologies in diet management and their effectiveness in anti-aging healthcare. After underlining the importance of nutrition in the aging process, we explored the applications of mobile apps, web-based platforms, wearables devices, sensors, the Internet of Things, artificial intelligence, blockchain, and other technologies in managing dietary patterns and improving health outcomes. The research further examines the effects of digital dietary control on anti-aging healthcare, including improved nutritional monitoring, personalized recommendations, and behavioral and sustainable changes in habits, leading to an expansion of longevity and health span. The challenges and limitations of digital diet monitoring are discussed, and some future directions are provided. Although many digital tools are used in diet control, their accuracy, effectiveness, and impact on health outcomes are not discussed much. This review consolidates the existing literature on digital diet management using emerging digital technologies to analyze their practical implications, guiding researchers, healthcare professionals, and policy makers toward personalized dietary management and healthy aging.

## 1. Introduction

Aging, a slow but steady process defined by a progressive deterioration in the normal physiological functioning of living organisms during their lives, is often closely related to various epigenetic mechanisms that help in the regulation of the aging journey [1] and have a significant role in the onset and progression of age-related diseases. The manipulation of these epigenetic mechanisms has helped develop some scientific processes and the subsequent development of various medical interventions for anti-aging healthcare. However, most anti-aging treatments concentrate on menopausal and andropause symptoms, which are hormonal alterations that do not necessarily reduce regularly, making such interventions relatively difficult; this highlights the need for other modes of intervention [2]. Anti-aging healthcare incorporates a range of medical and lifestyle practices aimed at preventing, slowing down, or reversing the processes associated with aging [3]. Seeking suitable anti-aging healthcare requires appropriate interventions such as nutrition, constant medical checkups, exercise, skincare, and stress management, all promoting healthy aging and improving an individual’s quality of life [4]. The world population is progressively aging, and the growth of the world’s older population will continue to outpace that of the younger population over the next 35 years [5]; hence, the need for comprehensive approaches that address the multifaceted aspects of aging and prioritize preventive measures arises.

Traditionally, some common practices have promoted a healthy aging process by slowing down or mitigating the effect of aging on the human body; these practices help improve a person’s overall condition and facilitate a healthy aging process [6,7]. A balanced diet of sufficient quantities of essential nutrients (water, carbohydrates, fats, proteins, vitamins, and minerals) and phytochemicals that support cellular health has proven to be one of the best practices for maintaining good health conditions, thus forecasting a healthy aging process [8]. However, estimating the standard nutrient intake that is necessary for an individual requires some knowledge that could necessitate an expert consultation. Visiting a nutrition and dietetics specialist for customized recommendations is often advisable. Regular exercise has also been promising in promoting a healthy aging process. Aerobic exercises, strength training, and flexibility exercises help maintain healthy cardiovascular conditions, boost mood, and reduce stress. Smoking and excessive alcohol consumption are associated with various health risks that negatively affect the aging process; moreover, uncontrolled sugar intake and processed foods are causes of inflammation and oxidative stress [9]. Therefore, regular health checkups are a good practice for the early detection of an abnormal condition, followed by an appropriate treatment plan. In addition, stress management, adequate sleep, and body hydration also contribute to a healthy aging process. Adopting the above practices does not guarantee a healthy anti-aging process, but it certainly contributes to its achievement. Though the overall success of anti-aging measures is physically visible through appearance, especially through how the skin looks, it is important to note that skincare products mainly contribute to keeping the human skin hydrated with fewer wrinkles, thus reducing chronological aging from a physical appearance perspective [10]. Some novel approaches have been developed to help monitor the above practices to ensure a perfect follow-up and maintain a delay in biological aging. Most of the successful ones make use of digital technologies [11]. 

Due to the non-invasiveness, versatility, and speed of anti-aging processes, digital anti-aging mechanisms appear to be more prevalent. Digital anti-aging refers to reversing or delaying the aging process using digital products, resulting from technological advancements, to promote a healthy aging mechanism. Digital technologies play a crucial role in improving anti-aging healthcare [12]. These technologies offer several benefits, including personalized interventions for diagnosis and treatment, telemedicine and remote monitoring, efficient data collection, enhanced accessibility of health information, and artificial intelligence-driven diagnosis [13]. By leveraging digital tools, individuals can actively participate in managing their health, track their progress, and receive real-time feedback. Integrating IT tools in anti-aging interventions can empower individuals, improve adherence to healthy habits, and facilitate early detection and intervention in age-related health issues. More so, digital technologies such as artificial intelligence (AI), blockchain (BC), the Internet of Things (IoT), immersive technologies, and digital twins open up new opportunities for anti-aging healthcare [14,15]. Additionally, the current metaverse, a recent platform for immersive interactions, can potentially revolutionize the anti-aging process, thus promoting a healthy aging life [14]. By leveraging emerging technologies, digital anti-aging healthcare can provide new healthcare experiences, reduce healthcare costs, improve patient outcomes, and create personalized treatment agendas to improve the anti-aging process. 

Digital technologies have emerged as powerful tools in healthcare, offering innovative solutions for monitoring and managing various aspects of health and well-being. This research focuses on the impact of these digital technologies in managing diet as part of anti-aging healthcare. This study’s main objective is to assess the effects of digital technologies on diet management and show their significance as a key factor in a healthy aging process. The contributions of this research are as follows:

- Raise awareness of the use of digital technologies for diet management.
- Demonstrate the impact of diet management and digital technologies in anti-aging healthcare.
- Improve anti-aging outcomes for the betterment of the aging population.

The paper aims to investigate the potential of digital technologies to control individuals’ diet and improve aging processes. Firstly, an overview, objectives, and contributions of the research are given in the introduction. Aging healthcare, involving the impact of aging on health and the role of nutrition in aging, is discussed in the second section. The third section examines the fundamentals of digital anti-aging healthcare, emphasizing its impact on monitoring and guiding dietary habits for anti-aging healthcare. Technological choices, user experience, and legal compliance in digital diet management are covered and the next section, which discusses the effects of digital diet control on anti-aging healthcare. The paper ends after highlighting the limitations, challenges, and future directions that can be adopted.

## 2. Aging Healthcare

### 2.1. Impact of Aging on Health

Aging is an inevitable biological process involving various physiological and functional changes in the body. Aging derives from many factors leading to a frailty state, characterized by general body weakness, increased risk of chronic disease, reduced physical and cognitive abilities, declines across several physiological systems, and overall decreased well-being. Aging is accompanied by changes, including transcriptional and epigenetics modifications, cell variations, and nuclei mechanics, that affect tissues, organs, and subcellular organelles, increasing the risk of chronic disease and death as time passes [16]. Age is a primary risk factor for most human diseases [17]. For example, cancers, cardiovascular diseases, diabetes, osteoporosis, neurodegeneration, and many other diseases present high risks in adults, followed by decreased cognitive function. Though aging can alter an individual’s physical appearance (wrinkles and gray hair), physiological changes can be affected by environmental factors such as diet, exercise, and exposure to toxins. Aging results in almost irreversible effects on the body’s physiology, including the immune system, with increasing vulnerability to infectious illness and contributing to aging-related cardiovascular, metabolic, autoimmune, and neurological disorders [18]. As the immune system declines with age, a substantial impact on the health and well-being of older people follows [19]. 

The physiological alterations during aging significantly affect the development of age-related diseases. This deterioration can result in a variety of age-related diseases, including Alzheimer’s, Parkinson’s, cardiovascular diseases, and cancer. The brain, in particular, is vulnerable to the effects of age, with age-related changes in the brain having a link to cognitive loss and dementia. Additionally, age-related alterations to the immune system can increase the likelihood of infections and cancer. The cardiovascular system also changes significantly with age; this increases the chances of having a heart attack or stroke. Moreover, age-related alterations to the musculoskeletal system can lead to a loss of bone density, muscular mass, and strength; this increases the probability of falls and fractures [20]. As we age, the immune system and the gut microbiome change significantly in composition and function, correlating with a greater vulnerability to infectious illnesses and decreased vaccine responses [21]. For example, oxidative damage contributes to the major hallmarks of aging. It is required in pathogenic pathways that are considered to induce a significant variety of age-related illnesses [22]. Subsequently, oxidative stress changes from the physiological level that is necessary for normal defense systems to a toxic level that is implicated in molecular or organelle damage, ultimately leading to various diseases [23]. Overall, the physiological changes during aging significantly impact the development of age-related diseases; this highlights the importance of prevention and early intervention.

### 2.2. Age-Related Diseases

The aging process is a dynamic, chronological process marked by the steady accumulation of cell damage, growing functional deterioration, and increased disease susceptibility and sensitivity [24]. Aging is always accompanied by numerous diseases, including cardiovascular disease (hypertension, stroke, and others), cancers (breast, prostate, colorectal, skin, and lung), neurological illness (Alzheimer’s disease (AD)), mental disorders, diabetes, etc. Various physical changes associated with aging include muscle mass loss, bone weakness or bone density reduction, and reduced flexibility, which may increase the likelihood of falls, fractures, and mobility difficulties [20]. A loss in muscle mass is also connected with an increase in fat mass and, as a result, changes in body composition and an increased prevalence of insulin resistance in older people [20]. For instance, with increasing age, there is a corresponding increase in the risk of developing type 2 diabetes, which is associated with senile skeletal muscle dysfunction and unregulated blood sugar levels due to a decreased sensitivity to insulin [25]. Additionally, mental and neurological illnesses account for 6.6% of total disability in older people [26]. Cognitive decline is another major effect of aging on health, mostly characterized by a substantial reduction in performance in mental tasks [27]. Furthermore, cancer-related diseases are often considered aging diseases, as the cancer incidence increases significantly after the age of 50. Anne and Shelley [28] discussed the responsibility of age dependency in cancers and underlined that age is one of the biggest risk factors for most cancers. Research indicates that dietary choices can equally influence certain risk factors associated with cancer development.

Moreover, nutritional frailty, a state of physical vulnerability or weakness associated with unfulfilled dietary requirements when needed by the body, is at the origin of most age-related diseases, resulting in decreased mobility and physical activity [29]. Frail individuals are exposed to conditions such as cardiovascular disease (CDV), cognitive decline, osteoporosis (bone disease), and sarcopenia (loss of muscle mass and strength) [30]. For example, CVD is mostly influenced by diets that are high in saturated fats and sodium. At the same time, inadequate calcium and vitamin D intake can lead to osteoporosis, characterized by weakening bones and associated with an increased risk of fractures. Ensuring that appropriate nutritional needs and hydration are satisfied and maintaining balanced diets are fundamental in reducing the risk of age-related diseases, thus living a healthy old age [31]. Older people are exposed to chronic obstructive pulmonary disease (COPD). They are advised to consume nutrient-dense foods to boost the immune system and make use of anti-inflammatory properties that are found in omega-3 fatty acids, which are abundant in fish and certain seeds [32,33]. Using digital technologies in diet monitoring appears to be the most promising solution, as it can be an effective and efficient way to ensure that personalized nutritional plans are provided and followed. 

### 2.3. Aging Biomarkers

Aging has a lot of implications for human health, which has caused variations between biological and chronological age [32]. For the past few decades, research has sought reliable biomarkers that indicate biological age rather than just chronology. These biomarkers are indicators that measure biological processes and are used to gain insight into aging and age-related diseases. Biomarkers are significant in objectively measuring biological processes that may not be observable using traditional methods in clinical practices. Telomeres are one of these biomarkers; they are protective caps at the end of chromosomes, shortening each cell division. A shorter telomere has been proven to be linked with some age-related diseases [33], notably CVD, AD, and diabetes. Other biomarkers associated with aging and age-related diseases include the epigenetic clock, oxidative stress, inflammation, and mitochondrial dysfunction [34]. The impact of biomarkers extends beyond their potential use in disease diagnosis and treatment. Biomarkers provide insights into the mechanisms underlying aging and age-related diseases and can also help identify individuals who are at an increased risk of developing these diseases. By analyzing biomarkers in large populations, researchers can better understand the factors contributing to healthy aging and develop interventions promoting healthy aging. For example, a study of more than 1000 people found that higher levels of the inflammatory marker C-reactive protein (CRP) were associated with a higher risk of developing cognitive impairment and dementia [35]. Another study found that high levels of homocysteine, a marker of oxidative stress, were associated with an increased risk of cardiovascular disease. These findings underline the possibility of biomarkers providing insights into the mechanisms of aging and age-related diseases [36].

Drew Liam [37] showed that the epigenetic clock was the most promising example of real biological age predictions. Biological markers are expected to track the condition of biophysical aging and give relevant insights into its underlying mechanism from molecular or phenotypic perspectives, for which digital applications have found great relevance [38]. Epigenetic clocks analyze changes in the Deoxyribonucleic acid (DNA) methylation patterns across the genome for real-age estimation. Nowadays, algorithms are used to calculate age based on their readout and certain health parameter checks. An adequate determination of biological age has several desirable advantages, in that it can be used for the appropriate selection of qualified individuals for early prevention of cardiovascular disease, and it also outlines points for appropriate behavioral, pharmacological, and other relevant interventions [32]. Subsequently, digital applications that can model senescence, predict the rate of aging, and monitor the processes and their human friendliness will help effectively evaluate and elucidate biological aging from chronological aging—a promising path for the early detection and management of several age-related diseases [37].

### 2.4. Potential Interventions in the Aging Process

Due to the complexity of age-related conditions, their prevention appears to be challenging. Biomarkers can provide significant information that can guide strategic interventions. Moreover, biomarkers help quantify biological processes, enabling accurate disease diagnostics, treatment, and prevention. Biomarkers reveal the underlying mechanisms of aging and age-related conditions, allowing for the development of interventions to improve health outcomes [39,40]. A set of biomarkers can predict the risk of developing CVD, diabetes, and cancers [34], thus helping to identify the individual at risk and suggest appropriate follow-up. While clinical practices consider constructing an index system to measure organ aging as a preventive measure for age-related diseases, digital interventions are promising for the elderly population. 

Evidence supports the importance of some practices for enhancing the overall health condition of persons aged 60 and above. For example, low-frequency, long-term, and frequent aerobic exercise is more helpful for mental disorders. Depending on their physical state, it is recommended that older people exercise at a reduced frequency [41,42], and for this, digital aerobic monitoring tools will be of immense use. Therefore, research is expected to focus on developing therapeutics that promote successful brain aging [43], for which digital anti-aging technology is relevant. Although intricate dynamics exist between changes in the brain’s structure and cognitive ability, technology-based interventions such as MRI (Magnetic Resonance Imaging) have helped improve the average life expectancy. Digital interventions are required for a close monitoring to help facilitate the achievement of the World Health Organization’s (WHO) “decade of healthy aging”, which is expected to span through 2020–2030 and focus on maintaining functional abilities during aging [44].

## 3. Role of Nutrition in Aging

Nutrition plays a crucial role, as it is a pivotal modulator of aging and a significant factor in adult health outcomes. The complex interactions between nutrients and cellular processes shape aging and age-related diseases. The quantity of nutrients and phytochemicals in a food determines its health benefits. Nutrients are molecules that are required by the body for its proper growth and maintenance. Nutrients include water, carbohydrates, fats, proteins, vitamins, and minerals, which are essential for the adequate functioning of the body. Phytochemicals or phytonutrients are bioactive compounds in plants. By exerting antioxidant, anti-inflammatory, and anti-cancer effects, phytochemicals are believed to improve human health. Appropriate nutrition, involving adequate and balanced food intake, is essential in maintaining optimal body function, supporting immune system function, maintaining muscle mass and strength, and preventing or alleviating chronic disease [45]. Moreover, consuming specific nutrients and adhering to specific dietary habits affect cellular metabolism, oxidative stress, inflammation, and the regulation of cell signaling pathways associated with aging [46]. For example, caloric restriction and certain dietary components such as antioxidants, polyphenols, and omega-3 fatty acids have been shown to potentially modulate aging-related cellular processes, including mitochondrial function and autophagy [47]. Nutritional factors have been proven to amend age-related cellular and molecular processes such as genomic instability, telomere attrition, epigenetic changes, and cellular senescence [47,48]. Nutrient-sensing pathways and nutritional interventions have emerged as promising targets for modulating aging characteristics and potentially delay the onset of age-related diseases [49]. The effects of diet on aging extend beyond cellular mechanisms, affecting organ function, systemic inflammation, and the development of age-related diseases [50]. A detailed understanding of how dietary components interact with aging-related biological processes is critical to elucidating the mechanisms of healthy aging and developing targeted nutritional strategies to promote optimal health outcomes in older adults. Recent research has revealed that the metabolism plays a crucial role in controlling epigenetic changes, and that this regulation significantly impacts the aging process and longevity. Age-related cell differentiation reduces immune cell production for adaptation, leading to nutritional deficiencies such as anemia, malignancies, and genetic disorders [51,52].

Evidence suggests that a deficiency in vital nutrients leads to immune abnormalities that are seen with aging. Biomarkers help assess an individual’s nutritional status, providing valuable insights into the dynamic interplay between diet, aging, and health outcomes [53]. Biomarker assessments encompass a spectrum of indicators, including micronutrient levels, inflammation markers, metabolic parameters, and oxidative stress markers [54]. Micronutrient biomarkers such as vitamin D and B and minerals like zinc and magnesium are essential in maintaining psychological functions, thus accurately evaluating nutritional health conditions in the aging population [55]. Furthermore, inflammatory markers such as C-reactive protein (CRP) and interleukin-6 (IL-6) can provide insights into the inflammatory status associated with diet- and age-related chronic diseases [56]. In addition, oxidative stress biomarkers, including malondialdehyde (MDA) and glutathione peroxidase (GPx), reflect the balance between free radical production and antioxidant defense, thereby serving as a marker of oxidative damage and potential nutritional indicators of imbalance [57]. Sometimes, increased consumption of specific nutrients above recommended levels is required to maintain immune system function and lower disease prevalence in older people. Malnutrition in older people can exacerbate deficient immune function, infection susceptibility, and, ultimately, infection-related mortality and the inherent age-related reduction in immunological response [58]. Immunosenescence can be well modulated by proper and specific nutrition, such as supplementation with vitamin E above the recommended requirements in older adults, which can boost immune responses and increase the resistance to diseases and infections, zinc supplementation, probiotic supplementation, and increased consumption of polyunsaturated fatty acids (PUFAs), which are effective in aging nutrition [59]. Assessing an individual’s nutritional health status is fundamental to identifying targets for dietary intervention, which can significantly reduce health risks associated with aging. 

**Figure 1:**
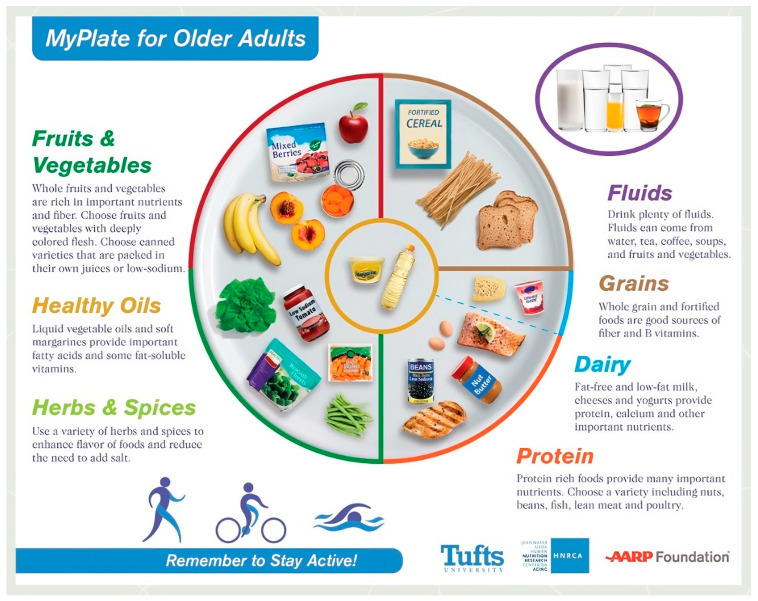
MyPlate for older adults [61].

A balanced diet is essential for good health and aging [59]. The aging population’s nutritional requirements differ from the standard requirements in adults, with a broad plate for older adults comprising a ratio of 50:25:25 for fruits and vegetables, whole grains, and protein-rich foods (Figure 1). Following MyPlate guidelines for adults, half of a plate should include fruits, vegetables, healthy oils, herbs, and spices that provide a variety of nutrients and fibers. One-quarter of the plate should be made up of whole grains and fluids that are rich in sources of vitamins and fiber. The rest of the plate includes multiple servings of fat-free and low-fat dairy products such as milk, yogurt, and cheese to get much-needed protein, calcium, and other nutrients. Smart eating becomes imperative as the body changes from young to old, requiring fewer calories, while the intestines begin to flow slower, increasing the risk of constipation [60]. Thus, it becomes critical to consume enough fiber. Moreover, dehydration is more common in older people, because they are less mindful of their bodies’ thirst cues. It is easy to forget to drink enough water when you are not thirsty, and increased fiber consumption raises fluid requirements as well, leading to the need for an increase in fluid intake [60]. Hydrating foods such as fruits and soups could also serve as potent alternatives. Adequate protein consumption is especially crucial for preserving muscular mass and strength, as muscle loss is a common phenomenon that progresses with aging.

As aging progresses, there is a reduction in the metabolic rate, usually with a corresponding requirement for a reduced caloric intake. However, calorie requirements vary from person to person depending on factors such as exercise level, muscle mass, and overall underlying health factors. Consequently, determining a suitable calorie intake for specific requirements may require the assistance of a healthcare practitioner or certified dietitian. Some of their interventions and services have also been well crafted into various digital tools and coaching, providing a wide range of options to boost versatility and aid in customizing these interventions. Based on specific health conditions, medications, and dietary preferences, a need arises for personalized nutrition to delay aging and protect the body from developing certain diseases. Several health monitoring applications for blood sugar, blood pressure, and the body mass index (BMI) now exist. Various online mobile applications directly provide relevant information and nutritional guidelines for older people based on the WHO’s standards for recommended daily intake (RDI). For instance, MyFitnessPal monitors caloric intake while helping users set realistic goals that can translate to visible health impacts. Waterlogging helps remind you to stay hydrated and track your water intake. HealthOut helps people find healthy meals while eating out based on various dietary requirements such as low-calorie, low-carb, low-fat, protein, and heart-healthy eating [53]. Consequently, in the future, there might be a need for the complete automation of diet screening and analysis for improved anti-aging interventions. 

## 4. Digital Anti-Aging Healthcare and Diet Management

### 4.1. Emergence of Digital Technologies in Healthcare

Traditional healthcare focuses on conventional methods such as physical and manual processing and the interpretation of healthcare data through in-person interactions between healthcare providers and patients, during which data from paper-based records and prescriptions, analog medical devices such as X-rays, and other equipment are used to perform diagnoses and treatment tasks. Traditional healthcare delivery services have gradually been digitalized, making them more effective and accessible. The healthcare industry has witnessed rapid growth through digital technologies, revolutionizing how healthcare is delivered and accessed [62]. Digital technologies are now mostly electronically based, requiring minimal processing time and energy. The advent of digital technologies in healthcare and the adoption of prospective solutions have transformed healthcare by improving communication, enhancing data collection and analysis, enabling remote monitoring, and facilitating personalized interventions. These technologies can potentially improve the effectiveness and efficiency of healthcare delivery, including anti-aging healthcare [63]. As more healthcare systems shift to digital solutions, the distinction between analog and digital healthcare technology becomes increasingly wide [64]. Digital technologies have several benefits in terms of efficacy, efficiency, accessibility, and data-analyzing capabilities [65], contributing to better patient care results. The positive impacts of digitalization on healthcare cannot be ignored, starting from the integration of Electronic Health Records (EHRs), which was a revolution in dumping paper-based records and enabling sharing of healthcare data, minimizing errors in diagnosis through accurate clinical decision support systems and smoothing population health management for the forecast of eventual diseases [65].

Furthermore, technological innovations have reduced in-patient care, promoting distance healthcare through telemedicine, allowing patients from remote areas to access medical experts, thus reducing travelling times and costs and accelerating the healthcare service delivery process. Telemedicine is mostly useful and sometimes inevitable during pandemic periods such as COVID-19 [66,67,68]. Supporting telehealth systems, the IoT has surfaced to primarily assist in collecting patient data and transmitting them to a server for analysis by experts or generating appropriate responses. In IoT systems, the patient is equipped with a device composed of sensors that is able to capture the clinical features to be analyzed. The captured values are transmitted to a server using a connector, and action may be taken accordingly [69]. These data help monitor chronic conditions, provide early warning signs, and facilitate preventive care [70].

On the other hand, artificial intelligence, through its subfields such as machine learning, deep learning, computer vision, and natural language processing, has revolutionized healthcare by enabling advanced data analytics, medical image analysis, disease diagnosis, and prediction of potential outcomes by using algorithms and a huge amount of data to learn and identify patterns within them [71]. The healthcare industry has witnessed the development of a multitude of personal health applications and remote monitoring devices to directly address the challenges related to healthcare facilities and the availability of service delivery, enabling on-demand and automated vital signs monitoring, medication tracking, and personalized health recommendations that are fundamental for effective and efficient self-management of potential diseases [72,73]. The interoperability of healthcare data and secure data transmission through Health Information Exchange (HIE) platforms has facilitated data sharing, thus enabling fast and reliable data analysis from a distance, reducing redundancies in data, and enhancing care coordination [74]. However, digital technologies have transformed healthcare delivery modes by expanding the landscape and improving outcomes. 

### 4.2. Digital Diet Management for Anti-Aging Healthcare

Effective anti-aging healthcare is needed as the population ages. Diet management has proven to be a fundamental aspect of anti-aging healthcare, as research demonstrates that maintaining a healthy diet prevents and delays most age-related diseases [75]. Diet management incorporates dietary planning, monitoring and tracking, nutrient intake analysis, diet personalization, nutritional goal setting, and behavioral change support. Traditional diet management methods, such as self-reported food diaries and 24 h recalls, are fraught with limitations and face several challenges, since they are time-consuming, inaccurate, and less effective due to their dependency on an individual’s memory and honesty [76]. One common challenge is the need for an electronic medical system that can effectively monitor dietary restrictions to assess claims that they can provide older adults with anti-aging benefits [77]. Other challenges include the complexity in estimating required nutrient intake and predicting accurate, personalized dietary challenges caused by humans’ genetic uniqueness, subjectivity, and compliance due to carelessness and forgetfulness, delays in assessing the impact of the process, etc. [78]. While traditional diet control methods face challenges, digital technologies provide new and innovative ways to handle diet, thus enhancing anti-aging healthcare outcomes. Digital technologies have the potential to revolutionize healthcare and anti-aging healthcare. Integrating digital technologies in the diet control process has emerged as a promising solution to address the challenges faced in the traditional setup [79]. Integrating digital technology into nutritional assessments improves data accuracy, streamlines the process, and reduces the participant burden. This has huge potential to advance public health initiatives, clinical research, and personalized nutrition plans. The most common evolution in diet management using digital technologies is mobile food-/diet-tracking applications [80]. These applications manage users’ data and use advanced analytic methods to generate recommendations, allowing healthcare providers to track and assess patients’ conditions [81]. They enable remote monitoring of patients through sensor-based accessories and IoT technology to monitor hydration, stress levels, and lifestyle to make informed decisions about diet and nutrition [82]. Other technologies, such as AI, blockchain, cloud computing, edge computing, 5G connectivity, nanotechnologies, point-of-care technologies, and more, are used in the backend or in combination with these applications to ensure their efficiency. For example, 5G connectivity eases data transmission from the wearable device to the data server [83]. At the same time, blockchains are used for food safety, a crucial factor in smart nutrition systems [84]. Anti-aging practices such as healthy dietary patterns, body mass index preservation, appropriate weight loss, wellness, and healthy lifestyles are significantly impacted by digital technologies [85]. 

#### 4.2.1. Mobile Applications for Diet Tracking (Food-Tracking Apps)

Diet-/food-tracking applications are the most common applications of digital technology in diet control. These applications’ main function is to allow users to keep track of their food intake and monitor their nutrient/calorie intake to generate personalized recommendations, which is essential to promoting healthy aging. IT developers and stakeholders, therefore, develop mobile applications that could serve the purpose of diet management. One of the most popular apps for food tracking is MyFitnessPal, which has a database of over 11 million food compositions, enabling the end user to track their food intake and monitor their calorie intake effectively. Using MyFitnessPal, a user can set goals for their diet, such as weight loss or muscle gain, and the application will provide personalized dietary recommendations. This app integrates a barcode-reading option to input new food items into the database quickly. Table 1 shows some popular examples, providing a more comprehensive picture of diet management apps. 

The above examples show that the United States of America (USA) has been leading the development of diet management apps for decades. Apart from its tech-savvy population, the USA has other reasons to lead this industry, including its health awareness, entrepreneurial culture, healthcare costs, focus on prevention, market demand, and environmental regulations. Most of these apps can be used for free with limited options; subscribing to a premium service requires a monthly payment, which leads to full access. Many other countries are also developing and adopting health and wellness apps. In Asia, research from Asian media research [86] conducted in 2021 during the COVID-19 pandemic shows that Malaysia, South Korea, Thailand, Indonesia, and Japan adopted health and wellness apps, including diet control apps. These Asian countries are developing solutions to meet the population’s demand through healthcare providers, stakeholders, and engineers. The Asia–Pacific diet and nutrition app market is expected to account for USD 4245.82 million by 2028 [87], led by Japanese, South Korean, Indian, and Chinese companies.

Although the apps mentioned above are mostly commercialized versions and off-the-shelf solutions for users, researchers in academia are also proposing promising mobile apps that encompass wider targets in anti-aging healthcare. Mortazavi et al. [79] reviewed the technological advances in diet monitoring and precision nutrition. The authors first focused on food photography and AI abilities to facilitate diet monitoring; secondly, they discussed physical sensors for detecting moments of dietary intake and chemical sensors for estimating the composition of diets and meals through wearable and handheld sensors that can potentially be used to automate certain aspects of diet logging. This research also discussed new programs to generate personalized/precision nutrition recommendations based on measurements of gut microbiota and continuous glucose monitors with AI. Furthermore, Divya et al. [88] researched diet monitoring and health analysis using artificial intelligence. The authors proposed a mobile app running a web-based chatbot using Naive Bayes classifiers. The proposed application categorizes diseases based on the user’s input and generates the diet plan that is needed for health improvement. The chatbots provide personalized nutrition plans and can use previous chats to have more meaningful conversations with users. Targeting a healthy lifestyle, Wang et al. [89] examined the impact of adherence to self-monitoring of one’s diet and physical activity in a technology-supported behavioral intervention on weight loss. Their research explored the use of various digital technologies, more precisely, mobile applications and wearables devices, for weight loss, while demonstrating the importance of consistent self-monitoring in achieving successful outcomes in weight management through technology-supported methods. Elina et al. [90] investigated factors related to the sustained use of free apps for diet monitoring to understand the factors influencing users’ ongoing engagement with mobile apps. By analyzing user behavior and interaction patterns, the research sought to identify key elements contributing to the app’s long-term adoption and sustained use for dietary tracking. The findings shed light on the app’s effectiveness and users’ motivations for consistently utilizing it in their dietary self-monitoring practices.

#### 4.2.2. Web-Based Platforms and Virtual Coaching

The above-presented mobile apps often provide a web-based platform that allows users to access it via a browser by requesting or receiving a response from a server. They operate on desktops and do not generally need to be installed as applications. Web-based platforms and virtual coaching have become increasingly popular in diet control. For example, MyFitnessPal and Noom provide web-based platforms like mobile apps that assist the user in conveniently and efficiently tracking their dietary habits and receiving personalized coaching from healthcare professionals and AI-based virtual chatbots. Such platforms facilitate social interaction and peer support through online forums and communities, fostering motivation and accountability in adopting healthy diets [91].

Another example is the Computer-Aided Nutritional analysis Program (CAN-Pro) [92], a Korean web-based dietary assessment and meal planning application. CAN-Pro incorporates an extensive database containing Korean foods and nutritional information, and it is often used for menu planning, dietary assessment, education, and research. On web-based platforms, individuals can get support and personalized advice from certified coaches from recognized organizations that give users daily tasks and challenges to achieve their dietary goals. Virtual coaching for diet control capabilities is further expanded through video conferencing platforms such as Zoom, Skype, and Google Meet for lively coaching sessions, with effective and confident virtual coaching boosting psychological satisfaction, leading to a healthy aging process.

#### 4.2.3. Wearable Devices, Sensors, and the IoT

**Figure 2:**
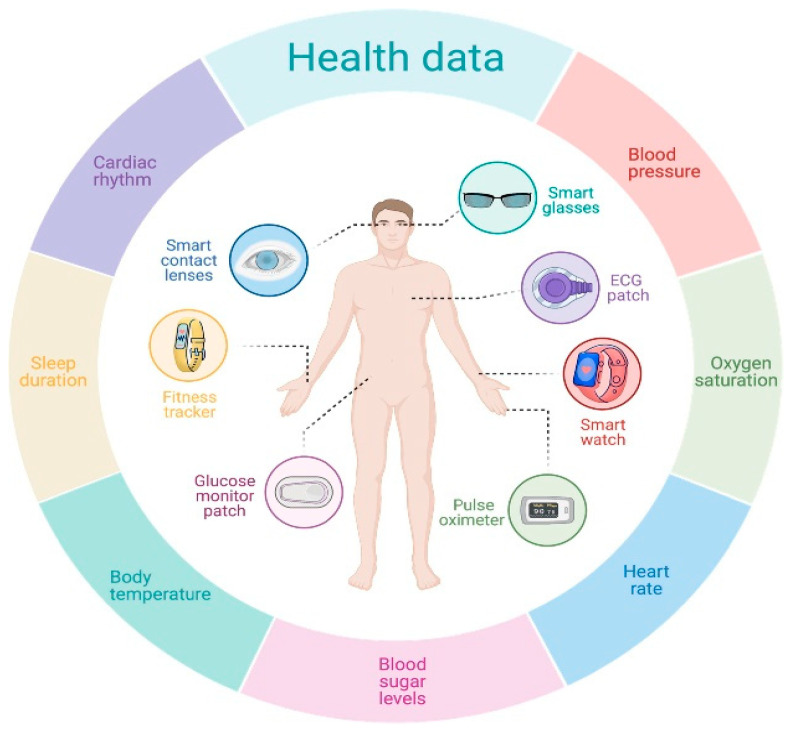
Wearable devices, attachable to the human body for dietary assessment [64].

In recent years, wearable devices equipped with sensors have played a vital role in effectively monitoring physiological parameters and capturing data related to eating behaviors [93]. Health professionals and patients use real-time feedback from wearable devices to track dietary intake conveniently and non-invasively. Figure 2 below shows a set of wearable devices and the position that they can occupy on the human body [64]. These devices include ECG (electrocardiogram) paths, smartwatches, and fitness trackers that can capture data related to physical activities, sleep patterns, and calorie expenditure. Some more sophisticated devices can detect chewing patterns, swallowing, and food intake, allowing for automatic tracking of eating habits [94]. Wearable devices and sensors like accelerometers, gyroscopes, and optical sensors enable real-time monitoring of physical activities and energy expenditure, facilitating decision making processes regarding dietary choices and thus allowing for personalized eating behaviors [95]. Another benefit of using wearable devices for diet management is the ability to track changes in dietary habits over time, allowing for the targeting of specific areas requiring improvement. The wealth of data from wearables provides precise measurement of food intake, portion sizes, and meal composition, facilitating personalized diet plans and recommendations.

Oliver et al. [96] presented an on-body sensing solution for automatic dietary monitoring. The researchers used electromyography (EMG) sensors, inertial measurement units (IMUs), and food intake sensors to automate dietary tracking in practice. Their study aimed to offer user-friendly and non-intrusive means of monitoring dietary intake to assist users in adhering to healthy eating practices and achieving their nutritional objectives. The potential outcomes of their study were discussed, highlighting that the proposed technology would promote healthier lifestyles and empower individuals to make well-informed decisions regarding their diet based on the data from the sensors. Similarly, Hassannejad et al. [97] examined the potential of wearable sensor-based methods and computer vision in automatic diet monitoring. Computer vision techniques entail the analysis of images and videos to recognize and monitor the food items that are consumed by individuals. At the same time, wearable sensor-based methods use wearable devices to gather data on dietary behaviors and patterns. Within this research, a comprehensive exploration of these methods’ advantages, limitations, and potential applications concerning the automated monitoring of individuals’ diets was conducted. 

As a collective network of connected devices and technologies that facilitates communication between devices and the cloud, IoT technology enables data transmission from sensor to server for further analysis. The IoT is widely used in diet tracking applications by combining real-time data on food intake with more accurate data from the user, enabling an assessment of their eating habits and thus facilitating effective decision making. KitchenMate is one of the most popular IoT-enabled diet-tracking apps and keeps track of user’s intake by connecting smart kitchen appliances to generate personalized recommendations based on set targets. IoT technology combines wearable devices to provide a more comprehensive picture of the user’s health. Juliane et al. [95] utilized wearable devices and mobile sensors to generate personalized nutrition plans. Their research explored digital technologies, including the IoT and cloud computing, to collect reliable data on dietary behavior and health patterns. The goal was to develop personalized nutrition solutions that empower users to make well-informed nutritional decisions, ultimately contributing to enhanced overall health and well-being. In terms of gender, Suganyadevi et al. [82] illustrated an innovative IoT-based diet monitoring healthcare system for women. The system includes a smart dining table with load sensors and RFID (Radio Frequency Identification) to track meal frequency and food intake. It also consists of a hybrid eating behavior monitoring device with a camera and microphone to detect portion sizes and intake. The fully automated system uses Wi-Fi-based sensors to assess the nutritional value of food and a smartphone app to collect nutritional information on food ingredients. It calculates the food’s weight and sends the data to the cloud for analysis. The system classifies foods based on their nutritional content and identifies nutrient deficiencies. At the same time, it tackles obesity by suggesting nutrient-dense, low-calorie food choices. The solution empowers users to make informed nutritional decisions and maintain a healthier lifestyle. Wearable devices, sensors, and the Internet of Things (IoT) have emerged as promising technologies for diet monitoring, contributing to anti-aging practices that are required for a healthy elderly age. 

#### 4.2.4. Artificial Intelligence

In finding applications across various domains, artificial intelligence aims to create systems and machines to perform human-intelligence-required tasks. AI solves complex problems by automating processes and making predictions using data. AI technology is increasingly integrated into healthcare for disease diagnosis, predictive and survival analysis, drug discovery, and personalized medicine [98]. One crucial aspect of personalized medicine is diet management, which is fundamental in anti-aging healthcare. AI algorithms can potentially analyze an individual’s dietary patterns, genetic data, personal preferences, health conditions, and targets to forecast a customized diet plan, thus optimizing nutritional interventions [99]. Table 2 below shows some popular datasets that are used to train the diet recommendation models for personalized nutrition. Most datasets are conceived using data from diet monitoring apps, wearables, and dietary reference intake (DRI). The DRI is a set of guidelines that helps individuals and healthcare professionals make informed decisions about nutrient intake. The DRI is a comprehensive set of nutrient reference values that have been scientifically developed for assessing and planning diets for well-being. The DRI is updated every five years due to changes in population characteristics, emerging health concerns, and policy and practice improvements. For instance, most US-based updated apps on Alexa use the DRI for Americans for 2020–2025. DRI guidelines encompass nutrient values, such as Adequate Intake (AI), Recommended Dietary Allowance (RDA), Estimated Average Requirement (EAR), and Tolerable Upper Intake Level (UL), which are fundamental for datasets and useful for AI model training and prediction of personalized diet plans [100]. Furthermore, a dataset contains information about dietary, clinical, demographic, biochemical, and anthropometric features; these data are analyzed to forecast personalized recommendations. In most cases, mobile applications use computer vision and object detection techniques, such as YOLO (You Only Look Once), SSD (Single Shot Detector), R-CNN (Region-Based Convolutional Neural Network), and HOG (Histogram of Oriented Gradients), to train food detection and classification models using food image datasets. In practice, a user captures an image of their meal using mobile apps. Computer vision-based algorithms handle food segmentation, and food regions can be analyzed using other AI algorithms and dietary datasets for recommendations or predictions. Dietary datasets provide sufficient information to inform about the nutritional value of a given food, allowing algorithms to generate personalized diet plans that fit the user’s goals. 

The data-driven approach enables AI to make use of various types of data to provide personalized recommendations, analyze dietary patterns, and support behavioral change. AI algorithms can facilitate personalized nutrition recommendations by analyzing dietary preferences, health goals, and nutritional needs to generate a customized meal plan considering other factors such as age, gender, weight, activity level, and dietary restrictions [112]. With the above image datasets, AI can use object detection algorithms on a photograph of the user’s meal to identify and estimate its nutritional content, allowing for accurate tracking of calorie intake, macronutrient distribution, and micronutrient levels [113]. With a focus on historical dietary data, AI algorithms can identify and predict future eating behavior, which is fundamental in dietary assessment and personalized nutrition schemes. Artificial intelligence has been integrated into smart kitchen appliances such as refrigerators, scales, and cooking utensils to optimize food preparation, monitor food inventories, and suggest recipes based on available ingredients. These techniques make it easier to stick to nutritional goals and promote effective meal planning [114]. Chatbots or voice-controlled interfaces provide on-demand guidance and support for nutritional management. Users can ask questions, receive personalized recommendations, track their progress, and set reminders to stick to their nutrition goals [115].

AI algorithms are used in the backend of mobile applications, and their existence seems to be invisible to the users. However, AI algorithms learn from specific data in datasets originating from appropriate data sources (DRIs, food frequency questionnaires (FFQs), 24 h dietary recall, food diaries, genetic data, wearables devices, and user feedback), adapting to personalized needs with continuous refinement of nutritional suggestions. Due to genetic and medical interindividual variability, AI is essential for handling complex analyses that can aid in identifying aging biomarkers, which is necessary to build robust models with the most significant features [116]. Some dietary research also incorporated AI to analyze the dietary content, timing, and long-term patterns of aging individuals accurately [117]. Anselma et al. [118] proposed an AI-based reasoning framework with a diet monitoring case study. Considering the challenges associated with healthy diet maintenance, the proposed framework tolerates some dietary transgressions by adjusting appropriate meals to help users recover from lapses. On the other hand, Begum et al. [119] investigated some methods that leverage AI to estimate food calories in diet assessment. The authors aimed to offer an accurate means to evaluate the caloric content of consumed foods using several algorithms that can generate a comprehensive and regular nutritional intake plan for individuals. Some more advanced studies used AI computer vision methods to scan and analyze images of food items using a mobile phone camera to evaluate their nutritional composition. For such model training, computer vision algorithms require large-scale images and labels representing multiple food items to express textures and lighting exposure from various angles. The main challenge in computer vision-based solutions is the data availability of a comprehensive set of annotated images. Kousik et al. [120] focused on the Ayurvedic diet system by developing a mobile app to scan food items, assess nutritional values, and provide personalized suggestions based on users’ predefined goals. Aslan et al. [121] proposed food segmentation algorithms using deep learning algorithms. The authors aimed to detect food regions in an image and measure the caloric composition. Supervised learning algorithms segment consumed food and non-food areas to evaluate the nutrient intake, thus optimizing dietary assessment. A visually based diet assessment (VBDA) architecture was explored by Wang et al. [122] with a focus on computer vision algorithms that were used to identify relevant dietary information from a given image automatically. This approach produced volume estimation and fine-grained food analysis. 

Other researchers explored methods, including natural language processing (NLP) and data mining [123,124], for virtual chatbots to recommend personalized diets and identify dietary patterns from text and unstructured mobile data. NLP can change how individuals record their food intake by turning text-based descriptions into nutritional insights. NLP algorithms can analyze and interpret food-related text, identifying specific foods, portion sizes, and cooking methods that are mentioned in descriptions or restaurant menus. This automated process reduces the burden on users, who may find it tedious to record food manually, and significantly increases the accuracy of the collected dietary data. NLP’s ability to derive meaningful insights from text-based nutrition data opens new avenues for research, healthcare, and personal nutrition management. It can detect dietary patterns, track nutrient intake, and identify potential allergens or intolerances that are mentioned in food descriptions. By making food diaries more accessible and user-friendly, NLP encourages more people to participate in recording their diet, which is critical for promoting healthier eating habits and managing chronic disease. In essence, NLP can revolutionize food logging by connecting text data and actionable dietary information to increase the accuracy, efficiency, and impact of dietary assessment for users. AI algorithms must be combined and interoperated with other technologies for effectiveness. George et al. and Juliane et al. [95,125] used AI to analyze data collected from wearable sensors and transmitted to a cloud server using the IoT. The users could reach well-informed dietary decisions based on real-time data, ultimately contributing to their well-being and thus ensuring a healthy aging process. By leveraging artificial intelligence, nutritional management systems can provide highly customized and precise guidelines for more effective dietary interventions in anti-aging health [126]. In addition, artificial intelligence identifies natural extracts with anti-aging properties and finds bioactive peptides in food, including anti-aging applications. With the advancement of these technologies, it is now possible to monitor an individual’s diet and provide them with personalized anti-aging healthcare.

#### 4.2.5. Blockchain 

Blockchain is a distributed ledger technology that enables the secure, transparent, and tamperproof recording of transactions [127]. Blockchain was created in 2008 by Satoshi Nakamoto, specifically to buy and sell Bitcoin and carry out financial transactions. Nowadays, blockchain finds applications in many fields, and its potential benefits across different sectors are numerous [127,128]. Blockchain can help increase efficiency, improve data security, and enhance patient privacy in the healthcare sector [129]. Likewise, blockchain is useful in anti-aging healthcare applications like diet management. Using blockchain technology in diet management, users can benefit from secure, transparent, and efficient systems for tracking food-related data throughout the supply chain. In this perspective, traceability, transparency, food safety and quality, increased effectiveness, and greater user satisfaction are achievable. Due to the decentralized and secure nature of the blockchain, it is possible to manage food safety issues, enabling real-time and accurate traceability of food products. When food products are tracked through their entire life cycle using blockchain, risks are significantly reduced, making the supply chain more efficient and thus enhancing food safety and quality. Blockchain technology can improve the privacy of nutritional data by using cryptographic principles and decentralized structures [130]. In addition, smart contracts in blockchain technology can be widely utilized in food supply chain systems to solve various food safety and quality issues [131]. By exploring the potential of blockchain technology, nutrition management can be transformed into a more effective and efficient system that supports individual health and well-being.

Blockchain technology can significantly impact diet management by tracing food from farm to table [132]. A trustworthy and reliable diet is achievable if the food source is authentic; therefore, consuming counterfeit food with unreliable nutritional descriptors will fail diet practice. Blockchain technology can effectively ensure transparency in the food supply chain, allowing consumers to check the products’ origin, nutritional values, and expiry dates. On the blockchain, smart contracts enable automated personalized dietary plans to help nutritionists and food advisers set specific nutritional intakes for their patients. Blockchain ensures transparency in changes and compliance for all records, and a token can be used to reward individuals who strictly follow the dietary guidelines to encourage healthy eating habits and promote their lifestyle. People with dietary restrictions such as allergies could benefit from the blockchain due to the possibility of tracking food information and reducing the risk of consuming risky items.

Furthermore, blockchain technology facilitates quality assessment, compliance with regulations, and consumers’ informed choices based on their preferences and dietary requirements. With blockchain, all activities associated with the supply chain, from farming to consumption, can be delegated to separate entities and traced back to the source. Tracking the food sources can follow the entire supply chain, and eventual problems can be identified and addressed swiftly, thus improving how people manage their diet. In addition, blockchain technology can guarantee the authenticity of food products. The in-built characteristics of blockchain, including its immutability and transparency, contribute to a dependable and trustworthy system for tracing food products. By utilizing a food traceability system on a blockchain platform, data collection can be guaranteed to be tamperproof, and real-time monitoring of livestock products during the supply chain can be accomplished. This decreases the probability of fraudulent activity, such as mislabeling and food fraud, which can threaten consumers’ safety and undermine trust in the food industry [133].

Recently, blockchain-based diet management platforms have gained popularity in response to concerns about food safety and quality affecting consumers’ health and the effectiveness of diet management. In general, these platforms provide a distributed ledger for all participants in the food supply chain, creating an immutable record and real-time view of all transactions between involved parties [134]. Although many functionalities are embedded in blockchain-based frameworks, effective diet management is achievable when the solution focuses on food safety and traceability, allowing the stakeholders to protect their businesses while establishing better risk management practices. Some famous blockchain-based solutions include:

- IBM Food Trust: A modular food traceability solution that is built using blockchain technology to operate across the food industry’s value chain [135]. IBM Food Trust provides real-time quality control, enabling increased visibility in the supply chain. Many businesses, including Walmart and Nestle, have adopted this platform, demonstrating significant potential for improving food safety.
- TE-FOOD: This solution provides a permissioned blockchain for every food organization, regardless of size. Through its blockchain layers, TE-FOOD enhances the security of food traceability, providing real-time quality control to guarantee consumer trust. Migros, one of Switzerland’s largest supermarkets, uses this platform to achieve end-to-end product traceability [136].
- Ambrosus: This platform uses blockchain to enhance patients’ trust and confidence in food products. Ambrosus has achieved end-to-end traceability and ensures food safety and quality [137]. It has been adopted by several businesses, including the Swiss coffee producer Lattesso and the French wine producer Château Latour.

However, the benefits of integrating blockchain in diet management can be summarized as follows: (1) increased transparency, accountability, and traceability, which are fundamental in addressing origin and quality concerns regarding food; (2) improved food safety and quality through a digital record of the food’s journey across the supply chain, ensuring that the food is safe, high-quality, and meets regulatory standards; (3) support for personalized nutrition and diet planning with a secure and efficient system to manage users’ data. Blockchain can be combined with other technologies to increase its capabilities. For instance, AI-driven technologies can be merged with blockchain for personalized dietary recommendations, enabling individuals to receive tailored nutrition plans that consider their unique factors and goals, improving public health outcomes. By leveraging the decentralization and security of blockchain, food intake monitoring can become more transparent, trustworthy, and personalized, allowing for more informed decisions about food choices and dietary intake assessment. 

#### 4.2.6. Other Technologies Involved in Digital Diet Monitoring

Other technologies can support a technical framework for diet in anti-aging healthcare. Earlier, we discussed the popularity of mobile apps, which appear to be the most used technology regarding diet monitoring. It could be observed that the full functionalities of these apps require the integration of other technologies depending on the goal. For instance, Gkouskou et al. [138] discussed using digital twin (DT) technologies in nutrition. The primary objective of the research was to investigate the potential benefits of a DT in offering more effective and personalized nutrition plans. The authors used a DT to create virtual representations of various nutrition-related processes, such as food intake and nutrient absorption within the human body. The researchers could simulate and analyze personalized nutrition patterns and individual requirements by integrating data analytics and machine learning. In addition, Mortazavi et al. [79] reviewed some advancements in digital technologies for diet management and precision nutrition. The authors covered the potential of mobile apps, AI, wearable devices, and sensors in easily generating personalized dietary recommendations considering gut microbiota measurements and continuous glucose monitors. Three-dimensional printing, a versatile technology that facilitates the customization and production of new product designs, was combined with AI and applied to food printing tasks. In a study by Bedoya et al. [139], the authors explored the possibility of integrating AI and 3D food printing technology to explore new protein sources for the food industry. This research shows how AI can analyze relevant data to optimize the use of these protein sources in food production. This study also investigates the application of 3D food printing in producing innovative and sustainable food products with specific nutritional profiles. The main goal was to gain an insight into the potential of combining AI and 3D food printing to develop novel and environmentally friendly food options that address future challenges in the food industry. Likewise, immersive technologies (virtual reality and augmented reality), 5G/6G connectivity, edge computing, and others can be integrated or used at the backend of some mobile applications to ensure developers’ and stakeholders’ goals. For instance, 5G/6G connectivity provides fast and reliable data transmission in digital diet management [83]. 

In addition, point-of-care (POC) technology provides devices and services to support clinical decision support and can equally extend its applications in anti-aging healthcare through diet monitoring [140,141]. POC devices are capable of continuously or periodically measuring a variety of age-related biomarkers such as B. blood glucose levels, inflammatory markers, oxidative stress indicators, and hormonal imbalances [142]. By providing real-time data on these biomarkers, individuals can gain insights into their metabolic health and make informed decisions about dietary and lifestyle changes. Moreover, POC devices can analyze biomarker data in conjunction with dietary intake information to create personalized nutritional recommendations based on individual needs and health goals [143]. Advanced machine learning algorithms and technologies can personalize nutritional recommendations based on factors such as age, gender, metabolic characteristics, and genetic predispositions, optimizing the effectiveness of nutritional interventions to achieve anti-aging aims. 

Furthermore, nanotechnology presents promising alternatives in certain aspects of healthcare, including anti-aging interventions through diet management. With its potential for targeted delivery of nutrients and precision medicine applications, nanotechnology can be combined with wearable sensors, data analytics, and personalized health platforms to optimize interventions to promote healthy aging through diet management [144,145]. Safaee et al. and Sherif et al. [146,147] have reported the development of nanosensors that are capable of detecting glucose levels, oxidative stress markers, and inflammatory cytokines. These sensors can be incorporated into wearable devices to provide continuous monitoring of metabolic status and physiological parameters. AI techniques can be used to analyze the collected data to generate personalized recommendations for diet and lifestyle management. The use of nanoparticles and liposomes to encapsulate nutrients and enhance their bioavailability has led to the innovation of nanocarriers for targeted nutrient delivery [148]. Nanocarriers ensure that nutrient absorption is carried out by the specific targeted tissue or cell, thus enabling precise nutrition and effective diet management. 

Such multi-technological integration demonstrates the significance of digital technologies in diet management and their positive impact on anti-aging healthcare.

## 5. Technological Choices, User Experience, and Legal Compliance in Digital Diet Management

Considering the above-presented digital technologies and their applications in diet management for anti-aging healthcare, there is a need for a combination of technologies to achieve some particular goals. This requires a knowledge-based approach that will enable the generation of possible options that are attainable through innovation or incremental changes. The technological choices in digital diet monitoring will influence the adjacent possibilities, reshaping the user experience, especially when considering norms and legal compliance. Technological decisions can be observed from different angles depending on the designer’s target. For example, data collection methods can be carried out using wearable devices, mobile apps, or manual data input methods, which can expand or limit the adjacent possibilities, thus affecting the granularity and type of available data. Integrating wearable devices into a system can ease biometric data collection (heart rate, sleep patterns, etc.), allowing for more personalized recommendations. Additionally, algorithm selection and analytical techniques significantly influence the eventual outcomes. For instance, advanced deep learning techniques can be associated with blockchain to uncover complex patterns in dietary data obtained from wearable devices or other external data sources (genetics data, nutritional databases, etc.) securely, leading to more personalized recommendations and enhancing the user experience. 

Achieving personalization and customization of recommendations can tailor individual preferences, dietary restrictions, and health goals, enhancing user engagement and satisfaction. The design of the user interface and the accessibility of diet monitoring platforms are crucial for ensuring a seamless and intuitive user experience; the choice of the platform’s architecture and the appropriate technologies is substantial and should be carefully made. Integrating user feedback mechanisms into these platforms can encourage adherence to dietary recommendation practices while enhancing user motivation and compliance [149]. 

Given the sensitivity of health and nutrition data, technology decisions must adhere to strict privacy regulations and security standards to protect user information. Implementing strong encryption protocols, access controls, and anonymity technologies can help ensure compliance with regulatory requirements and industry standards. Technology decisions should also consider ethical considerations around data use, consent, and potential bias. Transparent communication about collecting, processing, and using user data promotes trust and compliance with ethical standards, improving the overall user experience. Compliance with regulatory frameworks such as HIPAA (Health Insurance Portability and Accountability Act) or GDPR (General Data Protection Regulation) is critical for digital diet management platforms. Technology decisions should be consistent with these regulatory requirements to reduce legal risk and maintain user trust. 

In summary, technical decisions in digital diet management shape the possibilities of proximity by expanding the potential for personalized recommendations and insights and influencing the design of the user experience based on ethical and legal compliance considerations. In considering these decisions, developers can create innovative and user-centered solutions that empower individuals to make healthier dietary choices while respecting privacy and regulatory requirements.

## 6. Effects of Digital Diet Control on Anti-Aging Healthcare

The nexus of digital diet management and anti-aging healthcare generates a complex interplay system that opens realms where data-driven precision intersects with the pursuit of longevity. Understanding this integration and delving into its potential with thorough data analysis is fundamental for personalized anti-aging strategies, paving the way for significant insights, interventions, and informed decision making. The effects of digital diet management on anti-aging healthcare are discussed in four parts: improved nutrition monitoring and compliance, personalized diet recommendations, behavior change and sustainable habits, and the impact on longevity and health span.

### 6.1. Improved Nutrition Monitoring and Compliance

The advantages of digital tools for diet control have been demonstrated in anti-aging healthcare, illustrating their potential to enhance dietary monitoring and adherence. Such tools empower individuals to monitor their food consumption, track food intake, and establish dietary goals, cultivating greater mindfulness about their nutritional choices and nutrient intake [150]. Digital tools also provide real-time feedback and visual depictions of nutritional data, allowing individuals to monitor their progress and make informed decisions about their eating habits. According to research by Chen et al. [151], utilizing mobile applications for diet tracking can result in greater dietary self-monitoring and adherence to nutritional recommendations, ultimately leading to a better healthy lifestyle. Digital diet tools provide real-time tracking allowing users to track their nutritional intake in a real-time setting using wearable devices, IoT, sensors, and point-of-care technology. AI and machine learning techniques can facilitate nutrient analysis, and considering the user’s goal, these methods can generate a personalized nutrition plan that improves nutrition and compliance. 

### 6.2. Personalized Diet Recommendations

Diet personalization provides opportunities to improve people’s quality of life [152]. It can be categorized into three major categories: The first is personalized nutritional recommendations supplied through digital technologies, mostly mobile phone applications and other supporting techniques. The second level of customized dietary guidance will contain phenotypic information on anthropometry, physical activity, clinical parameters, and nutrition. Genetic data will be used in the third level of customized nutrition [153]. The future direction of personalized nutrition for healthy aging will incorporate more digital tools into the phenotypic and genomic levels of customized nutrition for improved anti-aging interventions. Using digital technologies, users can achieve personal goals based on individual characteristics and preferences. AI facilitates the analysis of giga data and provides recommended dietary solutions [126]. Unique nutrient requirements, dietary restrictions, and allergies can be accurately suggested for individuals, thus resulting in targeted monitoring facilitating well-being that is supported by better dietary adherence and improving anti-aging outcomes. 

### 6.3. Behavior Change and Sustainable Habits

Digital nutrition monitoring tools aid in behavior change and establishing sustainable nutrition habits for age-defying health. These tools facilitate self-monitoring, accountability, and motivation through features such as goal setting, progress tracking, reminders, and educational resources [154,155]. Digital diet monitoring helps people better understand their eating habits, enables them to make healthier choices, and promotes sustainable habits. Sustainability is achieved through a consistent routine and behavioral pattern change driven by a targeted intervention and personal goals. Digital tools can help individuals make gradual and sustainable changes to their eating habits by providing real-time feedback and personalized advice. Mobile apps and web-based diet management platforms can lead to positive behavioral changes, including an increased intake of fruits and vegetables, reduced intake of unhealthy foods, and overall improved diet quality [156]. By allowing for self-monitoring, goal setting, and cognitive restructuring, through integrated technologies, digital diet platforms can ensure sustainable habits that will promote a healthy lifestyle.

### 6.4. Impact on Longevity and Health Span

Although the direct impact of digital diet management on longevity and health is still undergoing research and evaluation, it can be observed that these tools have the potential to impact aging healthcare outcomes positively. Digital anti-aging healthcare tools can help extend an individual’s health span by promoting healthy eating habits, optimizing nutritional status, and supporting overall health. Long-term adherence to healthy eating habits, supported by digital tools, can help reduce the risk of age-related chronic diseases, improve physiological functions, and promote healthy aging [157]. Dietary control systems such as calorie restriction have been linked to enhanced metabolism, longer life spans, and a delay in developing age-related illnesses [158]. It also plays a major role in activating immune cells. Digital tools can determine how eating habits might affect life expectancy. For instance, the Food4HealthyLife calculator [159], a decision-support model that predicts how nutrition affects lifespan, helps doctors, policy makers, and laypeople understand the health implications of dietary choices [160]. Even though prior digital interventions on lifespan expansion results have some limitations, digital assessment tools can help reduce the limits of regular assessment methods by being simple and cost-effective. 

## 7. Challenges and Limitations in Digital Diet Management

**Figure 3:**
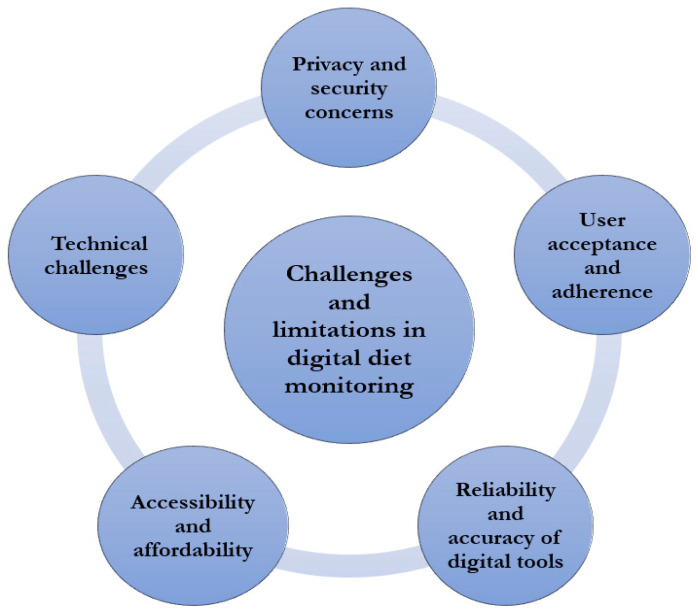
Challenges and limitations in digital diet monitoring.

Although digital diet control presents many benefits for older people, some challenges can be observed during its effective deployment and use (see Figure 3). 

### 7.1. Privacy and Security Concerns

Digital tools such as mobile applications are vulnerable in terms of privacy and security in the healthcare environment. Digital tools enable a lot of data processing, including personal data (food preferences, health conditions, and eating habits), which are a target to intruders who will try to gain access illegally and demand rigorous safeguards. Data breaches, unauthorized access, or misuse of sensitive information are potential risks to personal data. Though most users do not fully understand the privacy implications of using digital tools for diet management, they should have full control of the collected data. Cyber threats like hacking, malware, and ransomware can compromise data confidentiality and integrity. Addressing these concerns is imperative to safeguarding user privacy and ensuring user trust and confidence in the digital environment [161,162]. Robust privacy policies, compliance with data protection regulations, and data encryption are critical to handling these concerns. Even though we presented the blockchain as a means to ensure security in the food supply chain for diet control, it should be noted that blockchain has demonstrated its capability to secure individuals’ data effectively and thus can be used accordingly in nutritional settings [163]. 

### 7.2. User Acceptance and Adherence

User acceptance and compliance are key challenges in using digital diet control tools in anti-aging health. Not everyone may be comfortable or familiar with using digital technologies, especially older adults, who may face barriers to technology literacy, accessibility, and usability [164]. Motivating users to consistently use digital tools and adhere to recommended nutritional interventions can also be challenging. User-friendly and visually appealing interfaces are more likely to be adopted and used consistently by users, providing appropriate training and support, and tailoring interventions to individual needs and preferences can help improve user acceptance and compliance [165]. Another factor that can enhance user acceptance and adherence to digital diet monitoring platforms is their perceived utility and effectiveness, which will convince the user by showing them how useful the product can be for them. The user’s acceptance of digital diet monitoring services can be enhanced through meal tracking, goal setting, and progress monitoring. 

### 7.3. Reliability and Accuracy of Digital Tools

The reliability and accuracy of digital nutritional management tools in anti-aging health are important considerations. While these tools provide convenient real-time monitoring, there may be limitations in data collection and analysis accuracy. Errors, misinterpretations of data, and algorithm differences in nutrition tracking can impact the reliability of the information that is provided to users [166]. For example, AI algorithms used for nutritional assessment can be biased. If not carefully developed and tested, these algorithms may perpetuate existing health disparities, because they are based on historical data that may reflect biases in health, care, or eating habits. Ensuring that AI systems are trained on diverse and representative datasets is critical to prevent unfair or inaccurate results. Continuous monitoring and testing of AI algorithms to identify and correct biases must be a priority to promote fairness and impartiality in nutrition assessments. Digital tools must undergo rigorous testing and validation to ensure accuracy and reliability in providing nutritional advice and feedback.

### 7.4. Accessibility and Affordability 

The accessibility and affordability of digital diet management tools pose challenges to their widespread adoption and use. Not all individuals have access to smartphones, portable devices, or stable internet connections, limiting their ability to benefit from these tools. Additionally, cost barriers associated with purchasing and maintaining digital devices or subscribing to premium features and services may restrict access for certain populations [167]. To ensure equitable access to digital nutrition management tools for anti-aging health, accessibility and affordability issues must be addressed. Some common affordability strategies include free or low-cost options, reducing financial barriers for individuals with limited financial resources. Freemium models that offer basic features for free while providing premium features through paid subscriptions allow the user to enjoy basic functionality at no cost, thus increasing their adherence and accessibility to the platform. Subsidized programs and community and non-profit initiatives, are other alternatives that can boost the accessibility and affordability of digital diet monitoring platforms. 

### 7.5. Technical Challenges

Technical challenges associated with digital diet monitoring include several key aspects that impact the accessibility, usability, and overall effectiveness of these tools. These challenges often revolve around integration and compatibility issues, including device compatibility between different platforms and operating systems, limiting the accessibility and usability for users switching between devices. Additionally, software updates or version differences may affect functionality and data synchronization. Technical limitations, such as reliance on a consistent internet connection for real-time data updates, can be challenging, especially in areas with poor connectivity. Moreover, older or less advanced devices may find it difficult to run certain monitoring applications, negatively impacting the user experience.

## 8. Future Directions and Recommendations

Several factors will guide the future of digital diet management. Firstly, digital technology advancements must be considered to enhance the potential of digital diet control in anti-aging healthcare. For instance, quantum machine learning methods can help powerful computers speed up dietary predictions and recommendations to generate personalized diet plans. Furthermore, the user experience can be improved through virtual reality and augmented reality due to the immersive experience that they offer. Additionally, federated learning techniques can help to safely use different kinds of health information without sharing them openly, which is significant in data sharing, ensuring the security and privacy of users. Using recent biosensors from advancements in nanotechnology and point-of-care devices, we can quantify aging biomarkers such as telomeres to enhance the anti-aging strategy and facilitate the accurate prediction of dietary plans according to a specific health condition. Secondly, integrating multiple data sources helps individuals understand their dietary and health status completely. Diversity in data sources (wearable devices, electronic health records, genetic testing, etc.) and thorough analysis result in a robust system with a holistic view of personalized and precise interventions for dietary recommendations. Lastly, the user experience should be enhanced to improve acceptability and adherence to digital diet management by proposing user-centric design principles, personalized feedback, and user-friendly interfaces. 

## 9. Conclusions

This research presents an overview of the applications of digital technologies in anti-aging healthcare, with a focus on diet management. Diet management tasks, including dietary planning, monitoring and tracking, nutrient intake analysis, diet personalization, nutritional goal setting, and behavioral change support, are highly impacted by the integration of digital technologies. Individuals can use digital tools to enhance their nutritional intake by tracking their foods, evaluating its nutrient composition, and setting dietary goals. This research shows that diet management mobile apps that are integrated with other supporting digital technologies are effective for diet control, making them a powerful tool for anti-aging healthcare. Although some challenges and limitations can still be observed in diet management practices while using digital tools, cost-effective and user-friendly solutions can enhance the user experience, thus improving the acceptance and adherence of individuals to such solutions. As a result, this study presents possibilities for extending lifespans and ensuring longevity through digital diet management. 

## Figures and Tables

**Table 1:** Some popular diet-monitoring mobile apps.

No.	App Name	Description	Country	Year	Cost
1	MyFitnessPal	This app tracks food and calorie intake with a large database, barcode scanner, recipe importer, restaurant logger, and food insights.	United States	2005	Basic: Free Premium: (USD 9.99/month)
2	Lose It	Lose It simplifies your weight loss process with tools like a recipe library, nutrition log, and calorie counter. The app collects information about your height, weight, age, and specific goals to create a personalized plan tailored to your needs.	United States	2008	Basic: Free Premium: (USD 4.17/month)
3	Noom	Noom offers customized meal plans, weekly challenges, and a team of virtual coaches. It provides educational information, tools for tracking your progress, and training plans to incorporate more activities into your daily life.	United States	2008	Basic: Free Premium: (USD 60/month)
4	HealthifyMe	The app provides a comprehensive list of recipes and meal plans. Lifesum offers a barcode scanner and macro tracking to view your daily meals and calories.	India	2012	Basic: Free Premium: (USD 12/month)
5	YAZIO Fasting & Food Tracker	A popular and effective tool for nutrition tracking that allows users to monitor their food intake, track nutrients, and manage their fitness goals.	Germany	2013	Basic: Free Premium: (USD 100/year)
6	FatSecret	It offers a food diary, a large food database, a training diary, a weight chart and diary, and tons of healthy recipes to support your efforts. The app also features food image recognition, making logging and tracking calories easier than ever, and a unique meal calendar that visually shows when you are eating and burning the most calories.	Australia	2007	Basic: Free Premium: (USD 9.49/month)
7	Fastic	The app is designed to help users with intermittent fasting and offers features like tracking fasting times, providing meal plans, and providing educational content about intermittent fasting and healthy living.	Germany	2019	Basic: Free Premium: (USD 11.99/month)
8	MyNetDiary	MyNetDiary is a mobile app and online platform designed to help individuals track their diet, manage their weight, and achieve their health and fitness goals. The app incorporates an easy-to-use calorie counter that facilitates digital diet assistance for weight loss.	United States	2007	Basic: Free Premium: (USD 18.95/month)

**Table 2:** Personalized nutrition data sources for diet recommendations.

No.	Data Source	Description	Data Type	Ref.
1	USDA National Nutrient Database	Provides information on food and nutrients in various subsets: Food and Nutrient Database for Dietary Studies (FNDDS), National Nutrient Database for Standard Reference Legacy (SR Legacy), USDA Global Branded Food Products Database (Branded Foods), and Experimental Foods.	Anthropometric, demographic, biochemical, clinical, and dietary	[101]
2	Global Dietary Database (GDD)	Contains vast amount of original survey data and metadata, with vital information on dietary patterns. The metadata allow the user to select their data interest by choosing the desired diet methodology, country of interest, year range, sex, age, dietary variables, etc.	Demographic, clinical, and dietary	[102]
3	European Food Information Resource (EuroFIR)	EuroFIR contains food composition and nutritional information across Europe. It includes portion size and serving information, quality control validation, and metadata documentation.	Demographic, clinical, and dietary	[103]
4	Food Images Dataset	Food-5k contains 2500 × 2 food and non-food images for image classification. Food-11 has 16,643 food images grouped into 11 major food categories. Other datasets include Food-101, Caltech-256, RagusaDS, UEC-FOOD100, UEC-FOOD256, UEC-FoodPIX, and the Pittsburgh fast food image dataset	Images, annotations, mask images, and dietary data	[104,105,106,107,108]
5	National dietary surveys	NHANES in the United States or the National Diet and Nutrition Survey in the United Kingdom contains data on various health and nutrition indicators, including dietary intake, physical activity, and biomarkers.	Demographic, clinical, and dietary	[109]
6	National Institutes of Health (NIH)	NIH provides a dietary supplement label database of products marketed in the USA, including ingredients’ dosage information	Demographic, clinical, and dietary	[110]
7	KNHANES	Korea National Health and Nutrition Examination Survey contains health-related information such as demographics, health behaviors, healthcare utilization, physical measurements, biochemical and laboratory tests, dietary intake, and socioeconomic status.	Demographic, clinical, and dietary	[111]
8	CAN-Pro	The Computer-Aided Nutritional analysis Program is a nutrient database in Korea. It describes commonly consumed Korean food’s composition in a revised edition, with the latest nutritional composition per specific portion of food, which is useful for dietary assessment and menu planning.	Dietary, menu	[92]

## Data Availability

Not applicable.
